# The Importance of Marine Predators in the Provisioning of Ecosystem Services by Coastal Plant Communities

**DOI:** 10.3389/fpls.2018.01289

**Published:** 2018-09-03

**Authors:** Trisha B. Atwood, Edd Hammill

**Affiliations:** Department of Watershed Sciences and Ecology Center, Utah State University, Logan, UT, United States

**Keywords:** trophic cascades, top-down control, vegetated coastal ecosystem, blue carbon, mangroves, tidal marshes, kelp, seagrass

## Abstract

Food web theory predicts that current global declines in marine predators could generate unwanted consequences for many marine ecosystems. In coastal plant communities (kelp, seagrass, mangroves, and salt marsh), several studies have documented the far-reaching effects of changing predator populations. Across coastal ecosystems, the loss of marine predators appears to negatively affect coastal plant communities and the ecosystem services they provide. Here, we discuss some of the documented and suspected effects of predators on coastal protection, carbon sequestration, and the stability and resilience of coastal plant communities. In addition, we present a meta-analysis to assess the strength and direction of trophic cascades in kelp forests, seagrasses, salt marshes, and mangroves. We demonstrate that the strength and direction of trophic cascades varied across ecosystem types, with predators having a large positive effect on plants in salt marshes, a moderate positive effect on plants in kelp and mangroves, and no effect on plants in seagrasses. Our analysis also identified that there is a paucity of literature on trophic cascades for all four coastal plant systems, but especially seagrass and mangroves. Our results demonstrate the crucial role of predators in maintaining coastal ecosystem services, but also highlights the need for further research before large-scale generalizations about the prevalence, direction, and strength of trophic cascade in coastal plant communities can be made.

## Introduction

**FIGURE 1:**
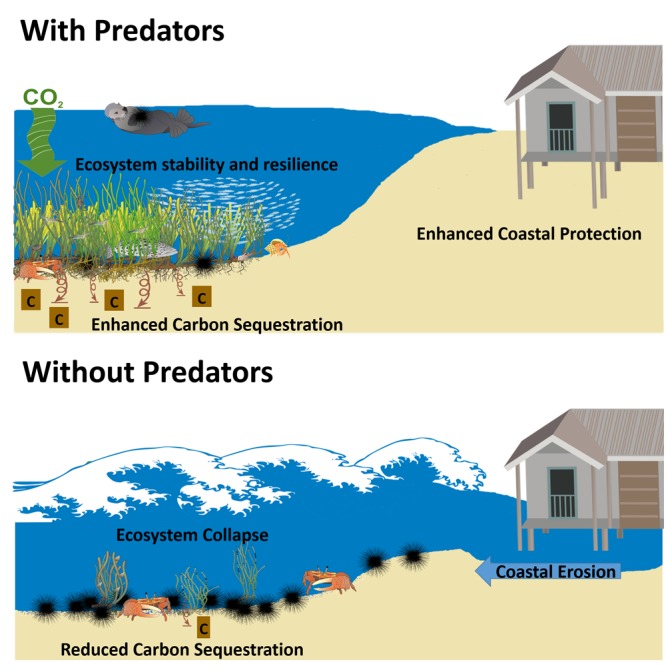
Predicted effects of predators, or lack thereof, on ecosystem services (carbon sequestration, coastal protection, and ecosystem stability) in coastal plant communities. It is predicted that predators, through direct and indirect interactions with lower trophic levels, support increased carbon uptake in plants and soils, protect coasts from storm surges and flooding, and support stability and resistance.

The green world hypothesis predicts that the loss of predator control on herbivores could result in runaway consumption that would eventually denude a landscape or seascape of vegetation (Hairston et al., 1960). Since the inception of the green world hypothesis, ecologists have tried to understand the prevalence of indirect and alternating effects of predators on lower trophic levels (i.e., “trophic cascade;” **Figure 1**), and their overall impact on ecosystems (Estes et al., 2011). Multiple lines of evidence now suggest that top predators are key to the persistence of some ecosystems (Estes et al., 2011).

With an estimated habitat loss of >50%, coastal plant communities are among the world’s most endangered ecosystems (Zedler and Kercher, 2005; Waycott et al., 2009; Duarte et al., 2013). As bleak as this number is, the predators that patrol coastal systems have fared far worse. Several predatory taxa including species of marine mammals, elasmobranchs, and seabirds have declined by 90–100% compared to historical populations (Lotze et al., 2006; Paleczny et al., 2015). Interestingly, predator declines pre-date habitat declines (Lotze et al., 2006), suggesting that alterations to predator populations may be a major driver of change for coastal systems (Jackson, 2001; Jackson et al., 2012).

There is little doubt that collapsing marine predator populations results from overharvesting by humans. Localized declines and extinctions of coastal predators by humans began over 40,000 years ago with subsistence harvesting (McCauley et al., 2015). However, for most large bodied, marine predators (toothed whales, large pelagic fish, sea birds, pinnipeds, and otters) the beginning of their sharp global declines occurred over the last century, coinciding with the expansion of coastal human populations and advances in industrial fishing (Lotze et al., 2006; Lotze and Worm, 2009). Following global declines in marine predators, evidence of trophic cascades in coastal ecosystems started to emerge (Estes and Palmisano, 1974; Sala and Zabala, 1996; Myers et al., 2007; Heithaus et al., 2014), with the disturbing realization that they affected more than just populations of lower trophic levels (Estes et al., 2011).

Understanding the importance of predators in coastal plant communities has been bolstered by their documented ability to influence ecosystem services (**Figure 1**). Multiple examples have shown that changes to the strength or direction of predator effects on lower trophic levels can influence coastal erosion (Coverdale et al., 2014), carbon sequestration (Wilmers et al., 2012; Atwood et al., 2015), and ecosystem resilience (Hughes et al., 2016). The idea that the extirpation of predators can have far-reaching effects on the persistence of coastal plants and their ecosystem services has become a major motivation for their conservation in coastal systems (Estes et al., 2011; Atwood et al., 2015). Here, we discuss some of the effects of predators on coastal plant communities and the ecosystem services they provide. Although these examples provide evidence that the loss of predators has negative consequences for important ecosystem services, they do not give a sense of prevalence of trophic cascades in coastal plant communities. Furthermore, our examples highlight cases where predators had positive effects on the plant community, which in turn had a positive effect on ecosystem services. To determine whether our examples represent the rule for predator effects on ecosystem services in coastal plant communities or the exception, we conducted a meta-analysis to assess the strength and direction of trophic cascades in kelp forests, seagrasses, salt marshes, and mangroves.

### Coastal Protection

Coastal flooding and erosion are major threats to coastal areas, with the frequency and magnitude of such events expected to increase with climate change (Arkema et al., 2013). Protection by coastal plant communities has been identified as a relatively cheap and effective tool for mitigating the effects of coastal erosion and flooding (Arkema et al., 2013; Duarte et al., 2013; Möller et al., 2014). The aboveground structure of coastal plants attenuates wave energy, dissipating and reflecting it away from the shore (Coops et al., 1996; Koch et al., 2009; Möller et al., 2014; Rupprecht et al., 2017). In addition, the complex root structures of seagrass, salt marshes, and mangroves help trap and stabilize sediments, allowing shorelines to accrete, further attenuating wave energy and reducing coastal erosion.

Herbivores and omnivores can negatively impact the structure of coastal plant communities (Coverdale et al., 2014). Many types of marine herbivores consume the leaves, seeds/propagules, or roots of coastal plants with negative consequences for plant density and canopy height (Cargill and Jefferies, 1984; Heithaus et al., 2014). As the extent of vegetation is correlated with the degree of wave attenuation (Koch et al., 2009), such changes in the structure of coastal plants could significantly reduce their ability to attenuate wave energy.

Predator declines have been implicated at least in part, in the collapse of several coastal plant communities, reducing coastal protection (Silliman et al., 2005; Coverdale et al., 2012). For example, recreational fishing of predatory marine crabs and fish along the east coast of the USA has relaxed predation pressure on sesarmid crabs. In response to lower predator abundance, sesarmid crab densities have increased six-fold and their burrows can cover up to 90% of the surface area of some marshes (Coverdale et al., 2012). These burrows undermined the structural integrity of the marsh, causing >10 cm of horizontal erosion annually, effectively removing >150 years of coastal accretion <30 years (Coverdale et al., 2014). These results suggest that reinstating top-down control in degraded coastal plant communities could help alleviate coastal flooding and erosion. However, further studies linking trophic cascades to changes in coastal protection are needed, especially in vulnerable areas like Florida, California, and New York where coastal habitats provide the greatest risk reduction from coastal hazards (Arkema et al., 2013).

### Enhance Carbon Sequestration

Kelp forests, seagrass, salt marsh, and mangroves are among the world’s most productive ecosystems, with global net primary production rates of 0.01–0.64 Pg carbon yr^-1^ (Duarte et al., 2013). In addition to storing carbon in plant biomass, seagrasses, salt marshes, and mangroves also store a significant amount of carbon in their soils (McLeod et al., 2011; Fourqurean et al., 2012; Duarte et al., 2013; Atwood et al., 2017; Macreadie et al., 2017b). With carbon turnover rates that are an order of magnitude slower than terrestrial soils, coastal wetlands represent the ultimate sink for otherwise rapidly cycled carbon (McLeod et al., 2011). Although kelp does not accumulate large soil carbon deposits, kelp forests are carbon donors, exporting carbon to shelf and deep sea sediments (Krause-Jensen and Duarte, 2016; Filbee-Dexter et al., 2018).

Predators can shape the structure of coastal plant communities through consumptive (lethal) and non-consumptive (risk) effects on herbivorous prey, altering the storage of carbon in plant biomass (Griffin et al., 2011; Christianen et al., 2014). The return of sea otters to the North American west coast revived overgrazed kelp forests, increasing carbon captured by kelp by upward of 8.7 Tg (Wilmers et al., 2012). Conversely, declines in other marine predators along the California coast allowed epiphytes to smoother the leaf surface of seagrass, reducing photosynthetic rates and dropping seagrass production by 50% (Lewis and Anderson, 2012). Although in both the above cases the negative effects of predation on herbivory had positive effects on plant growth, herbivory is an important process in coastal plant communities. Under natural or low levels, grazing can stimulate primary production by encouraging new growth (Cargill and Jefferies, 1984). This suggests that a delicate balance in herbivory, which can be accomplished through predation, is needed to ensure maximum productivity.

Not only do predators protect carbon sequestration in plant biomass, they also increase carbon sequestration in coastal soils (Atwood et al., 2015, 2018; Macreadie et al., 2017a). For example, a 50% reduction in the density or canopy height of macrophytes has been shown to increase sediment resuspension 10-fold (Gacia et al., 1999). Predators can moderate the effects of herbivores on coastal plant communities by significantly reducing their consumption of plant biomass and reducing the spatial extent of herbivory (Griffin et al., 2011; Atwood et al., 2015). Such cascading effects of predators has been shown to increase carbon accumulation rates and belowground carbon stocks in seagrass, salt marshes, macroalgal systems, and to a lesser extent mangroves (Atwood et al., 2015, 2018).

### Promote Ecosystem Stability and Resilience

Over the past 50 years we have lost 20–50% of global seagrass, salt marsh, and mangrove ecosystems (Zedler and Kercher, 2005; Duarte et al., 2013). For kelp the story is more variable, with region-specific changes that reflect both increases and decreases in kelp forest abundance (Krumhansl et al., 2016). Declines to coastal plant communities can be attributed to a multitude of interacting stressors (e.g., rising water temperatures, sea level rise, and eutrophication), many of which are anthropogenically driven. In the face of so many disturbances, stability and resilience may be the key to ensuring ecosystem persistence.

A few natural experiments have provided evidence that predators serve an important role in the stability of coastal plant communities. In Elkhorn Slough, an estuary in California, nutrient loading from agriculture led to a sharp decline in eelgrass in the area between 1965 and 1984 (Hughes et al., 2013). The recovery of eelgrass in the estuary in 1985 and again in 2005 coincided with the return of sea otters. Sea otters generated a four-tiered trophic cascade that ultimately resulted in the reduction of epiphytes, which reduce seagrass growth through shading (Hughes et al., 2013, 2016). Although rare, experiments investigating the ecosystem-level effects of predator recovery represent one of the few ways that we can examine their influence on ecosystem stability/resilience. This is because in many cases marine predator populations are already severely depleted (Dulvy et al., 2014; Paleczny et al., 2015), and their role in ecosystems altered.

A re-occurring issue with quantifying how predators impact ecosystems is that measures of stability and resilience are inherently multifaceted (Donohue et al., 2016). One of the goals of understanding stability and resilience is to aid in the recovery of ecosystems, which in itself is a multifaceted problem. Managers tasked with restoring ecosystems by promoting predators should establish baseline data, and set clear measurable targets (e.g. “restore plant biomass to 75% and sediment retention to 50% of historical levels”). Only through quantifying recovery targets can the impacts of disturbances associated with the loss of predators be quantified, and mitigated (Donohue et al., 2016).

## The Occurrence of Trophic Cascades in Coastal Plant Communities

The body of literature documenting trophic cascades in coastal plant communities provides opportunities to investigate variability in trophic cascade strength, direction, and persistence. We used a meta-analysis to assess and compare the strength and direction of trophic cascades in kelp forests, seagrasses, salt marshes, and mangroves. We collected data from publications on trophic cascades in coastal plant communities by searching the following terms in Scopus and Google Scholar in 2017 and through cross-referencing: predator^∗^ OR consumer^∗^ OR “trophic cascade” OR top-down OR grazer^∗^ OR herbivor^∗^ AND “salt marsh” OR “tidal marsh” seagrass OR mangrove OR kelp OR “coastal wetland.” We retained studies that included all of the following (1) predator effects on herbivore abundance, biomass, or foraging intensity (e.g., bite rates), (2) herbivore effects on plant biomass, abundance, or leaf damage, and (3) reported the mean values of the data, sample size, and some measure of variance for both herbivore/grazer and plant data. To summarize our results we used data from 68 field and lab studies (31 kelp, 23 salt marsh, 5 seagrass, 5 seagrass epiphytes, and 4 mangrove). Studies used in the analysis can be found in the **supplementary information**. When results were reported as a time series, we used the final sampling event. Although studies that crossed predator manipulations with other treatments (e.g., temperature, nutrients) can help predict potential changes in predator effects under future change, we focused our analyses solely on predator effects because crosses were often confounded with multiway interactions. In seagrass, several studies exist where the effects of herbivore exclusion on seagrasses were measured, but the link between predators and herbivores was only inferred (Heithaus et al., 2014). We did not include these studies in our meta-analysis because they did not directly measure the effect of predators on herbivores/grazers. Effect sizes were calculated using Hedges’ g, a commonly used effect size metric that corrects for small sample sizes. Hedges’ g effects sizes were quantified using the following equation:

$$\begin{array}{c} Hedges^{\prime }g=\frac{M_{p}-M_{A}}{SD_{\mathrm{pooled}}^{*}} \end{array}$$

where *M*_P_ was the mean of the herbivore or plant response in the presence of predators, *M*_A_ was the mean of the herbivore or plant response in the absences of predators, and $SD_{\mathrm{pooled}}^{*}$ was the pooled standard deviation.

**FIGURE 2:**
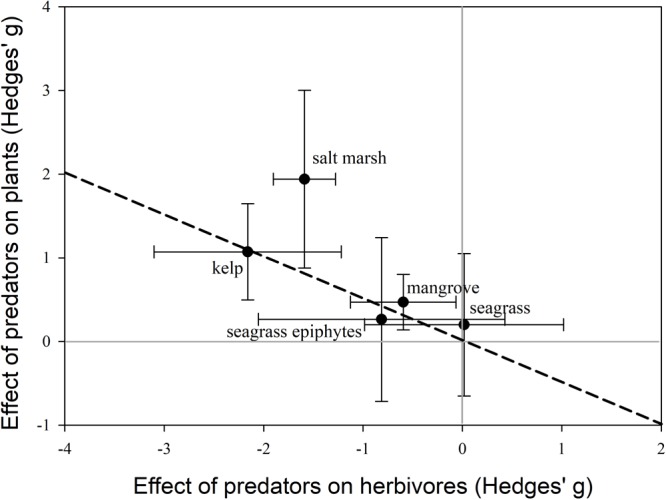
Comparison of trophic cascade strength for kelp, salt marshes, mangroves, seagrass, and seagrass epiphytes. Effect sizes (Hedges’ g ± 95% confidence intervals) represent the effect of predators on herbivores/grazers vs. primary producers. The effect of predators on the response is significant if the 95% CI does not overlap zero, zero for both the y-axis and x-axis are indicated by the light gray lines. The larger the effect size on primary producers the stronger the trophic cascade strength. The dotted line represents the 1:1 relationship.

For mangroves, salt marshes, and kelp, the average effect of predators on coastal plants was positive (**Figure 2**), suggesting that predators are likely to promote carbon storage, ecosystem stability and resilience, and reduce coastal erosion in these systems. However, in all four ecosystems, cases existed where predators had negative impacts on coastal plants; although these studies generally examined the effects of intermediate predators. Interestingly, in seagrass systems the effects of predators on consumers and seagrass and seagrass epiphytes were highly variable, and not significantly different from zero. This could suggest that globally, trophic cascades may be relatively weak in seagrass systems. In terms of trophic cascade strength, predator effects on primary producers was greatest in salt marshes followed by kelp and then mangroves (**Figure 2**). This could suggest that ecosystem services in these three systems are more susceptible to changes in predator populations, while ecosystem services in seagrasses are less vulnerable.

By examining the characteristics of the available trophic cascades studies in coastal plant communities (**Supplementary Table S1**), we identified research bias that constrained our ability to make broad generalizations about how changes to the strength or direction of predator effects will influence these systems. First, studies on seagrass and mangroves were extremely limited. This suggests that either the scientific community lacks interest or funding for studying trophic cascades in these systems, or there is publication bias. If there is a tendency to not publish non-significant results, publication bias could be masking evidence that trophic cascades are not prevalent or are weak in these systems. The potential for publication bias is an artifact that plagues all meta-analyses. Second, 90% of trophic cascade studies in coastal plant communities were conducted in North America (∼65%) and Oceania (∼25%). Only a few studies that met our selection criteria were conducted in Europe (4) and Asia (3), and no studies were conducted in South America or Africa, which encompass the most heavily fished areas of the world (Kroodsma et al., 2018). Third, the study of large-bodied vertebrate herbivores and predators was limited. No studies looked at top-down effects on a vertebrate herbivore, despite well-known effects of sea turtles, sirenians, and fish on benthic primary production, especially in subtropical and tropical systems (Poore et al., 2012; Bakker et al., 2016). Although over half of our studies included the examination of vertebrate predators, such studies were completely missing for salt marshes and mangroves. Furthermore, few studies investigated the effects of top predators. Thus, the paucity of trophic cascade studies in coastal plant communities may stem from our inability to manipulate the most threatened group of predators, large-bodied coastal predators such as sharks, marine mammals and seabirds. The inability to conduct experiments that involve predator removals and additions also presents challenges for teasing apart the direct and indirect effects of predator losses, the importance of which has been demonstrated in macroalgal systems (O’Connor et al., 2013; Donohue et al., 2017). Fourth, if predators cannot be experimentally manipulated we must rely on the occurrence of natural trophic cascades. However, historical declines in top predator populations have already rendered them ecologically extinct in many systems. Without pre-decline data on system characteristics, we can only infer the importance of predators from correlations, anecdotal evidence, rare cases of predator recovery, or studies on the few remaining pristine coastal systems with healthy marine predator populations. Although these types of studies were more common for kelp forests, they were rare for seagrass, mangroves, and salt marshes, highlighting the need for long-term data set in these systems.

## Conclusion

Above we have discussed how predators can help protect the ecosystem services provided by coastal plant communities. However, our meta-analysis highlighted that the availability of studies in all four coastal plant systems is far below the volume needed to make broad generalizations about trophic cascades in these systems. Thus, the prevalence of top-down control in marine ecosystems is still debatable, especially for seagrass and mangroves. Furthermore, even if one concedes that top-down control is common in coastal plant communities, arguments about whether predators predominantly have positive or negative effects on plant communities remains an open question. Although marine predator conservation is important for multiple reasons (e.g., biodiversity), the argument that through top-down control they promote the persistence of coastal plant communities needs further study. However, this brings up a troubling question, if the science is not currently sufficient to make broad generalizations, can it get there in time to make a real contribution to our conversations about predator conservation in coastal communities? Continuing declines in marine predator populations suggest that we are running out of time to quantitatively “prove” to the world and ourselves that marine predators are important to the persistence of coastal plant communities and the ecosystem services they provide. This leaves coastal scientists with a conundrum. We can choose to wait, methodically building the evidence for or against predator effects on coastal plant communities, with a specific focus on increasing studies in seagrass and mangroves, underrepresented regions, and large-bodied predators and herbivores. However, conservation decisions must be made while there is still an opportunity to do so, otherwise the results of such studies will become obsolete (Martin et al., 2012). Our other option is to be more aggressive in our recommendations about predator conservation, despite our less than perfect knowledge about their effects in coastal plant communities.

## Author Contributions

TA designed the study and drafted the first version of the manuscript. TA and EH collected and analyzed the data. All authors contributed to the writing and editing of the manuscript.

## Conflict of Interest Statement

The authors declare that the research was conducted in the absence of any commercial or financial relationships that could be construed as a potential conflict of interest.
