# Supplementary material for: The Importance of Marine Predators in the Provisioning of Ecosystem Services by Coastal Plant Communities
Source: Front Plant Sci. 2018 Sep 3;9:1289. doi: 10.3389/fpls.2018.01289 (PMC6129962; doi:10.3389/fpls.2018.01289)
Supplement: Supplementary file 1 [file Table_1.DOCX]

**Supplementary information:**

Table S1: Characteristics of the studies used in our meta-analysis.

| **Study Characteristics** | **Total # of**  **studies** | **Total in kelp** | **Total in**  **salt marsh** | **Total in seagrass** | **Total in mangrove** |
| --- | --- | --- | --- | --- | --- |
| **Habitat type** | 68 | 31 | 23 | 10 | 4 |
|  |  |  |  |  |  |
| **Predator type** |  |  |  |  |  |
| Invertebrate | 33 | 8 | 20 | 1 | 4 |
| Vertebrate | 19 | 11 | 0 | 8 | 0 |
| Vertebrate & Invertebrate | 16 | 12 | 3 | 1 | 0 |
|  |  |  |  |  |  |
| **Herbivore type** |  |  |  |  |  |
| Invertebrate | 66 | 31 | 23 | 8 | 4 |
| Vertebrate | 0 | 0 | 0 | 0 | 0 |
| Vertebrate & Invertebrate | 2 | 0 | 0 | 2 | 0 |
|  |  |  |  |  |  |
| **Experiment type** |  |  |  |  |  |
| Manipulated | 31 | 4 | 18 | 5 | 4 |
| Natural | 27 | 25 | 0 | 2 | 0 |
| Natural & Manipulated | 10 | 2 | 5 | 3 | 0 |
|  |  |  |  |  |  |
| **Location** |  |  |  |  |  |
| Africa | 0 | 0 | 0 | 0 | 0 |
| Antarctica | 0 | 0 | 0 | 0 | 0 |
| Asia | 3 | 0 | 0 | 0 | 3 |
| Europe | 4 | 0 | 0 | 4 | 0 |
| North America | 44 | 14 | 23 | 6 | 1 |
| Oceania | 17 | 17 | 0 | 0 | 0 |
| South America | 0 | 0 | 0 | 0 | 0 |

**References used in the trophic cascade meta-analysis.**

Altieri, A. H., Bertness, M. D., Coverdale, T. C., Herrmann, N. C., and Angelini, C. (2012). A trophic cascade triggers collapse of a salt-marsh ecosystem with intensive recreational fishing. *Ecology* 93, 1402–10. doi10.1890/11-1314.1

Babcock, R. C., Kelly, S., Shears, N. T., Walker, J. W., and Willis, T. J. (1999). Changes in community structure in temperate marine reserves. *Mar. Ecol. Prog. Ser.* 189, 125–134. doi:10.3354/meps189125.

Baden, S., Emanuelsson, A., Pihl, L., Svensson, C. J., and Åberg, P. (2012). Shift in seagrass food web structure over decades is linked to overfishing. *Mar. Ecol. Prog. Ser*. 451, 61–73. doi:10.3354/meps09585.

Bertness, M. D., Brisson, C. P., Coverdale, T. C., Bevil, M. C., Crotty, S. M., and Suglia, E. R. (2014). Experimental predator removal causes rapid salt marsh die-off. *Ecol. Lett*. 17, 830–835. doi:10.1111/ele.12287.

Bologna, P. A. X. (2007). Impact of differential predation potential on eelgrass (*Zostera marina*) faunal community structure. Aquat. Ecol. 41, 221–229. doi:10.1007/s10452-006-9059-x.

Byrnes, J., Stachowicz, J. J., Hultgren, K. M., Randall Hughes, A., Olyarnik, S. V., and Thornber, C. S. (2006). Predator diversity strengthens trophic cascades in kelp forests by modifying herbivore behaviour. *Ecol. Lett*. 9, 61–71. doi:10.1111/j.1461-0248.2005.00842.x.

Coverdale, T. C., Bertness, M. D., and Altieri, A. H. (2013). Regional ontogeny of New England salt marsh die-off. *Conserv. Biol*. 27, 1041–1048. doi:10.1111/cobi.12052.

Davenport, A. C., and Anderson, T. W. (2007). Positive indirect effects of reef fishes on kelp performance: The importance of mesograzers. *Ecology* 88, 1548–1561. doi:10.1890/06-0880.

Davidson, A., Griffin, J. N., Angelini, C., Coleman, F., Atkins, R. L., and Silliman, B. R. (2015). Non-consumptive predator effects intensify grazer-plant interactions by driving vertical habitat shifts. *Mar. Ecol. Prog. Ser*. 537, 49–58. doi:10.3354/meps11419.

Denno, R. F., Gratton, C., Peterson, M. A., Langellotto, G. A., Finke, D. L., and Huberty, A. F. (2002). Bottom-up forces mediate natural-enemy impact in a phytophagous insect community. *Ecology* 83, 1443–1458.

Duffy, E. J., Richardson, J. P., and France, K. E. (2005). Ecosystem consequences of diversity depend on food chain length in estuarine vegetation. *Ecol. Lett*. 8, 301–309. doi:10.1111/j.1461-0248.2005.00725.x.

Edgar, G. J., Stuart-Smith, R. D., Thomson, R. J., and Freeman, D. J. (2017). Consistent multi-level trophic effects of marine reserve protection across northern New Zealand. *PLoS One* 12, 1–26. doi:10.1371/journal.pone.0177216.

Estes, J. A., and Duggins, D. O. (1995). Sea otters and kelp forests in Alaska: Generality and variation in a community ecological paradigm. *Ecol. Monogr.* 65, 75–100. doi:10.2307/2937159.

Estes, J. A., Tinker, M. T., Williams, T. M., and Doak, D. F. (1998). Killer whale predation on sea otters linking oceanic and nearshore ecosystems. *Science* 282, 473–476. doi:10.1126/science.282.5388.473.

Finke, D. L., and Denno, R. F. (2006). Spatial refuge from intraguild predation: implications for prey suppression and trophic cascades. *Oecologia* 149, 265–75. doi:10.1007/s00442-006-0443-y.

Finke, D. L., and Denno, R. F. (2005). Predator diversity and the functioning of ecosystems: the role of intraguild predation in dampening trophic cascades. Ecol. Lett. 8, 1299–1306. doi:10.1111/j.1461-0248.2005.00832.x.

Griffin, J. N., Butler, J., Soomdat, N. N., Brun, K. E., Chejanovski, Z. A., and Silliman, B. R. (2011). Top predators suppress rather than facilitate plants in a trait-mediated tri-trophic cascade. Biol. Lett. 7, 710–713.

Finke, D. L., and Denno, R. F. (2005). Predator diversity and the functioning of ecosystems: the role of intraguild predation in dampening trophic cascades. Ecol. Lett. 8, 1299–1306. doi:10.1111/j.1461-0248.2005.00832.x.

Ho, C. K., and Pennings, S. C. (2008). Consequences of omnivory for trophic interactions on a salt marsh shrub. *Ecology* 89, 1714–1722. doi:10.1890/07-1069.1.

Huang, A. C., Essak, M., and O’Connor, M. I. (2015). Top-down control by great blue herons *Ardea herodias* regulates seagrass-associated epifauna. *Oikos* DOI10.1111. doi:10.1111/oik.01988.

Hughes, B. B., Hammerstrom, K. K., Grant, N. E., Hoshijima, U., Eby, R., and Wasson, K. (2016). Trophic cascades on the edge: fostering seagrass resilience via a novel pathway. *Oecologia* 182, 231–241. doi:10.1007/s00442-016-3652-z.

Lafferty, K. D. (2004). Fishing for lobsters indirectly increases epidemics in sea urchins. *Ecol. Appl.* 14, 1566–1573. doi:10.1890/03-5088.

Lewis, L. S., and Anderson, T. W. (2012). Top-down control of epifauna by fishes enhances seagrass production. *Ecology* 93, 2746–57.

Marczack, L. B., Ho, C. K., Wieski, K., Vu, H., Denno, R. F., and Pennings, S. C. (2011). Latitudinal variation in top-down and bottom-up control of a salt marsh food web. *Ecology* 92, 276–281. doi:10.1890/10-1922.1.

McKay, K. M., and Heck, K. L. (2008). Presence of the Jonah crab Cancer borealis significantly reduces kelp consumption by the green sea urchin *Strongylocentrotus droebachiensis*. *Mar. Ecol. Prog. Ser.* 356, 295–298. doi:10.3354/meps07238.

Moksnes, P., Gullstro, M., Tryman, K., and Baden, S. (2008). Trophic cascades in a temperate seagrass community. *Oikos* 117, 763–777. doi:10.1111/j.2008.0030-1299.16521.x.

Moon, D. C., and Silva, D. (2013). Environmental heterogeneity mediates a cross-ecosystem trophic cascade. *Ecol. Entomol.* 38, 23–30. doi:10.1111/j.1365-2311.2012.01398.x.

Moon, D. C., and Stiling, P. (2003). The influence of legacy effects and recovery from perturbations in a tritrophic salt marsh complex. *Ecol. Entomol.* 28, 457–466. doi:10.1046/j.1365-2311.2003.00534.x.

Offenberg, J., Nielsen, M. G., Macintosh, D. J., Havanon, S., and Aksornkoae, S. (2005). Lack of ant attendance may induce compensatory plant growth. *Oikos* 111, 170–178.

Offenberg, J., Nielsen, M. G., MacIntosh, D. J., Havanon, S., and Aksornkoae, S. (2004). Evidence that insect herbivores are deterred by ant pheromones. *Proc. R. Soc. B Biol. Sci.* 271, S433–S435. doi:10.1098/rsbl.2004.0210.

Perez-Matus, A., and Shima, J. S. (2010). Density- and trait-mediated effects of fish predators on amphipod grazers: Potential indirect benefits for the giant kelp *Macrocystis pyrifera*. *Mar. Ecol.* Prog. Ser. 417, 151–158. doi:10.3354/meps08820.

Piovia-Scott, J. (2011). The effect of disturbance on an ant-plant mutualism. *Oecologia* 166, 411–420. doi:10.1007/s00442-010-1851-6.

Reisewitz, S. E., Estes, J. A., and Simenstad, C. A. (2006). Indirect food web interactions: Sea otters and kelp forest fishes in the Aleutian archipelago. *Oecologia* 146, 623–631. doi:10.1007/s00442-005-0230-1.

Salomon, A. K., Shears, N. T., Langlois, T. J., and Babcock, R. C. (2008). Cascading effects of fishing can alter carbon flow through a temperate coastal ecosystem. *Ecol. Appl.* 89, 1874–1887.

Schultz, J. A., Cloutier, R. N., and Côté, I. M. (2016). Evidence for a trophic cascade on rocky reefs following sea star mass mortality in British Columbia. *PeerJ* 4, e1980. doi:10.7717/peerj.1980.

Shears, N. T., and Babcock, R. C. (2002). Marine reserves demonstrate top-down control of community structure on temperate reefs. *Oecologia* 132, 131–142. doi:10.1007/s00442-002-0920-x.

Shears, N. T., Babcock, R. C., and Salomon, A. K. (2008). Context-dependent effects of fishing: Variation in trophic cascades across environmental gradients. *Ecol. Appl.* 18, 1860–1873. doi:10.1890/07-1776.1.

Silliman, B. R., and Bertness, M. D. (2002). A trophic cascade regulates salt marsh primary production. *Proc. Natl. Acad. Sci. U.S.A.* 99, 10500–5. doi:10.1073/pnas.162366599.

Silliman, B. R., Layman, C. A., Geyer, K., and Zieman, J. C. (2004). Predation by the black-clawed mud crab, *Panopeus herbstii*, in Mid-Atlantic salt marshes: Further evidence for top-down control of marsh grass production. *Estuaries* 27, 188–196. doi:10.1007/BF02803375.

Valentine, J. F., Heck, K. L., Blackmon, D., Goecker, M. E., Christian, J., Kroutil, R. M., et al. (2007). Food web interactions along seagrass-coral reef boundaries: Effects of piscivore reductions on cross-habitat energy exchange. Mar. Ecol. Prog. Ser. 333, 37–50. doi:10.3354/meps333037..

Watson, J., and Estes, J. A. (2011). Stability, resilience, and phase shifts in rocky subtidal communities along the west coast of Vancover Island, Canada. *Ecol. Monogr.* 81, 215–239. doi:10.1890/10-0262.1.
